# Philadelphia Notes

**Published:** 1894-08

**Authors:** 


### PHILADELPHIA NOTES.

From Our Special Correspondent.

How grateful must Buffalonians be for their pure Lake Erie water. The often spoken of supply of the City of Brotherly Love is and has been all Summer in such a condition that the local papers advise the citizens to have patience with the street sprinkler, because sprinkling is useless and he is obliged to spread the water with a shovel, it is so thick with Schuylkill mud.

Among the college circulars sent out about this time the Fourth Annual Announcement of the Philadelphia Post-Graduate School of Homeopathics deserves special mention. This is its fourth year. It has a teaching staff of *twelve*, but only *eleven* alumni, the result of three years' work, and out of these eleven, *six*, *i. e.*, every one who resides in Philadelphia and Camden, has received an appointment on the faculty as professors and instructors. The organon of Samuel Hahneman, edition of 1833, furnishes the sole groundwork of the principles taught. Just imagine teaching medicine in 1894 from a text-book sixty years old!

The Medico-Chirurgical College building, of Philadelphia, has had a narrow escape from destruction. The common and select councils had passed an ordinance providing for a boulevard from the city hall to the park, about a mile long and 100 feet wide, passing diagonally across blocks of buildings and public squares at a cost estimated variously from $6,000,000 to $15,000,000. This boulevard would have passed through the college grounds and hospital. Fortunately the mayor at the last hour vetoed the bill because all Philadelphia seemed to be up in arms against it, crying for better water supply and more school houses, which the councils, council-like, had ignored in their desire to beautify the city and make it "attractive to strangers." The mayor's action leaves the college buildings in the same old place, at least, for the present.

Dr. Geo. M. Gould has been very ill in London since July 1st with a severe attack of influenza and inflammation of the middle ear. He received the best of care at the house of Mr. Ernest Hart, editor of the *British Medical Journal*, and is now on the road toward recovery, hoping to be well enough to attend the meeting of the International Ophthalmologic Congress, at Edinburgh, on August 8th.

